# Classification of *NF1* microdeletions and its importance for establishing genotype/phenotype correlations in patients with *NF1* microdeletions

**DOI:** 10.1007/s00439-021-02363-3

**Published:** 2021-09-18

**Authors:** Hildegard Kehrer-Sawatzki, David N. Cooper

**Affiliations:** 1 Institute of Human Genetics, University of Ulm, Albert-Einstein-Allee 11, 89081 Ulm, Germany; 2 Institute of Medical Genetics, Cardiff University, Heath Park, Cardiff, CF14 4XN UK

## Abstract

An estimated 5–11% of patients with neurofibromatosis type-1 (NF1) harbour large deletions encompassing the *NF1* gene and flanking regions. These *NF1* microdeletions are subclassified into type 1, 2, 3 and atypical deletions which are distinguishable from each other by their extent and by the number of genes included within the deletion regions as well as the frequency of mosaicism with normal cells. Most common are type-1 *NF1* deletions which encompass 1.4-Mb and 14 protein-coding genes. Type-1 deletions are frequently associated with overgrowth, global developmental delay, cognitive disability and dysmorphic facial features which are uncommon in patients with intragenic pathogenic *NF1* gene variants. Further, patients with type-1 *NF1* deletions frequently exhibit high numbers of neurofibromas and have an increased risk of malignant peripheral nerve sheath tumours. Genes located within the type-1 *NF1* microdeletion interval and co-deleted with *NF1* are likely to act as modifiers responsible for the severe disease phenotype in patients with *NF1* microdeletions, thereby causing the *NF1* microdeletion syndrome. Genotype/phenotype correlations in patients with *NF1* microdeletions of different lengths are important to identify such modifier genes. However, these correlations are critically dependent upon the accurate characterization of the deletions in terms of their extent. In this review, we outline the utility as well as the shortcomings of multiplex ligation-dependent probe amplification (MLPA) to classify the different types of *NF1* microdeletion and indicate the importance of high-resolution microarray analysis for correct classification, a necessary precondition to identify those genes responsible for the *NF1* microdeletion syndrome.

**Supplementary Information:**

The online version contains supplementary material available at 10.1007/s00439-021-02363-3.

## Introduction

Neurofibromatosis type 1 (NF1; MIM#162200) is one of the most common inherited cancer predisposition syndromes with an estimated frequency of 1:3000 (Lammert et al. 2005). Among all patients with NF1, 5–11% of patients have large deletions encompassing the entire *NF1* gene and its flanking regions at 17q11.2 (Cnossen et al. 1997; Rasmussen et al. 1998; Kluwe et al. 2004; Pasmant et al. 2015; Zhang et al. 2015). These ‘*NF1* microdeletions’ are often associated with a severe clinical manifestation of NF1 causing the *NF1* microdeletion syndrome (MIM#613576). Considered as a group, *NF1* microdeletion patients often have a more severe form of NF1 as compared to patients with intragenic pathogenic *NF1* variants. However, a certain degree of variability in terms of clinical symptoms has been observed on an individual level when comparing different patients with *NF1* microdeletions. These clinical phenotypic differences are likely to be caused by various factors including differences in deletion size and hence the number of genes co-deleted with the *NF1* gene. Four types of large *NF1* deletion (type-1, 2, 3 and atypical) have been identified that are distinguishable in terms of their size and breakpoint location, by the number of genes located within the deletion region and by the frequency of somatic mosaicism with normal cells lacking the deletion. Somatic mosaicism with normal cells is likely to cause a milder disease manifestation in patients with *NF1* microdeletions as compared to patients with germline *NF1* microdeletions.

**Fig. 1:**
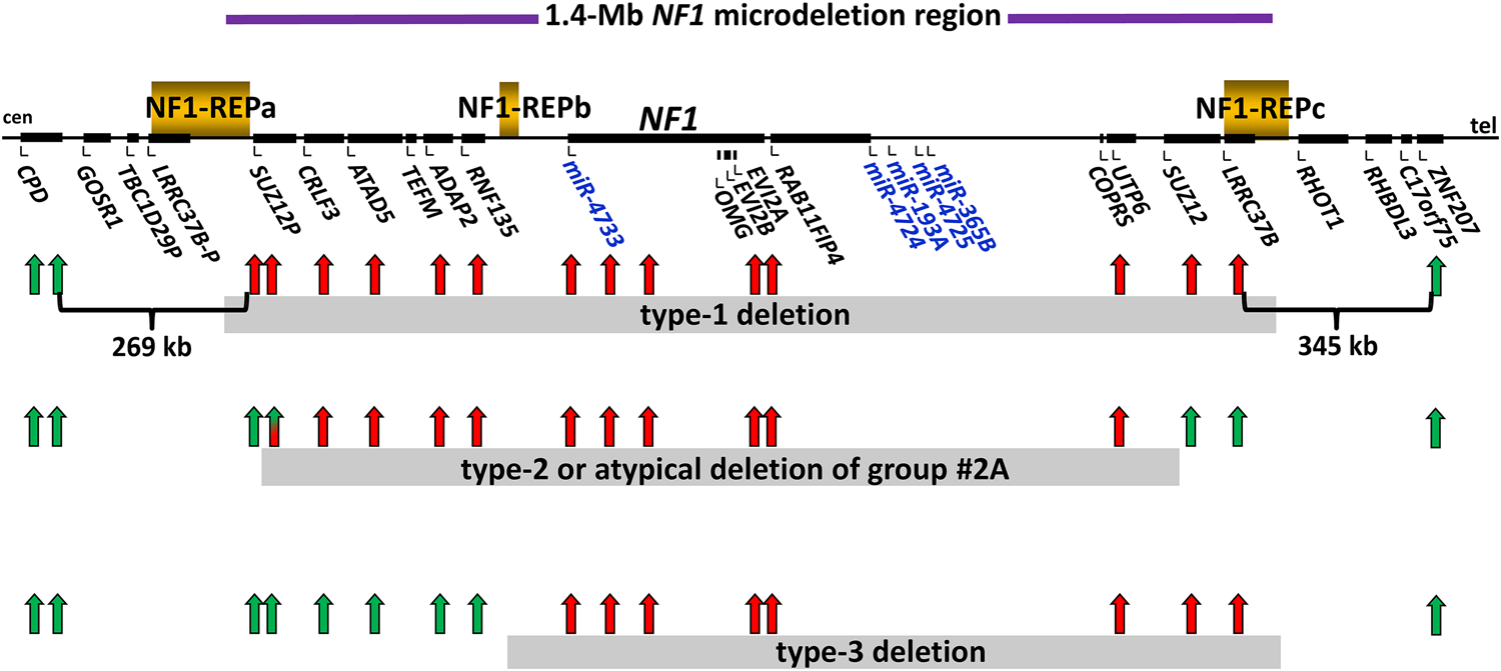
Schema of the 1.4-Mb spanning type-1 *NF1* microdeletion region and its flanking regions indicating the relative positions of the 14 protein-coding genes, the *SUZ12P* pseudogene and the 5 microRNA genes located there. The relative extent of type-1, type-2, atypical deletions of group #2A and type-3 *NF1* deletions are indicated by grey horizontal bars. The vertical red and green arrows represent the binding sites of the MLPA-probes included in the SALSA^®^ MLPA^®^ Probemix P122-D2 NF1-area (MRC-Holland). Red arrows represent MLPA-probes targeting genomic regions encompassed by the respective deletions whereas green arrows represent MLPA-probes targeted to regions which are not deleted and present in two copies. For example, in case of type-1 *NF1* deletions encompassing 1.4-Mb, the target sequences for the probes shown in red are present in only one copy because they are located within the deletion region. By contrast, the target sequences for the MLPA-probes shown in green are present in two copies since they are not located within the deletion region. The MLPA-probe *SUZ12P* intron 4 is shaded in green and red because the region targeted by this probe is deleted in some but not all type-2 and atypical group #2A *NF1* deletions. Type-2 deletions and atypical group #2A deletions cannot be distinguished by means of MLPA. *cen* centromeric; *tel* telomeric

Most frequent among all *NF1* microdeletions are the type-1 *NF1* deletions which encompass 1.4-Mb and include 14 protein-coding genes as well as five microRNA genes (Fig. 1) (Dorschner et al. 2000; Jenne et al. 2001; López-Correa et al. 2001). Type-1 deletions account for 70–80% of all large *NF1* deletions and usually occur as germline lesions that are present in all cells of the affected patients (Messiaen et al. 2011; Summerer et al. 2018). Most type-1 *NF1* deletions are caused by interchromosomal non-allelic homologous recombination (NAHR) during maternal meiosis (López-Correa et al. 2000; Neuhäusler et al. 2018). The NAHR events causing type-1 *NF1* deletions are mediated by the low-copy repeats, NF1-REPa and NF1-REPc. Within these low-copy repeats, recurrent breakpoints have been detected within two NAHR hotspots, termed paralogous recombination sites 1 and 2 (PRS1 and PRS2) (Forbes et al. 2004; De Raedt et al. 2006; Bengesser et al. 2014; Hillmer et al. 2016, 2017).

In contrast to type-1 *NF1* deletions, type-2 deletions encompass only 1.2-Mb and are associated with hemizygosity for 13 protein-coding genes since the *LRRC37B* gene is not located within the type-2 *NF1* deletion interval (Fig. 1). It has been estimated that 10% of all *NF1* microdeletions are type-2 (Messiaen et al. 2011). NAHR between the *SUZ12* gene and its pseudogene *SUZ12P* is the cause of the vast majority of type-2 *NF1* deletions (Vogt et al. 2012). In contrast to type-1 deletions, type-2 *NF1* deletions are frequently of postzygotic origin, mediated by mitotic NAHR, and hence are associated with somatic mosaicism of normal cells without the deletion (Kehrer-Sawatzki et al. 2004; Steinmann et al. 2007; Roehl et al. 2010, 2012). It has been estimated that at least 70% of all type-2 deletions are mosaic with high numbers of cells (94–99%) harbouring the deletion in blood, but with lower proportions in skin fibroblasts (39–91%) and urine cells (24–82%) (Steinmann et al. 2007; Messiaen et al. 2011; Roehl et al. 2012). Type-3 *NF1* deletions are rare, occurring in only 1–4% of all patients with *NF1* microdeletions. They encompass 1-Mb and are mediated by NAHR between NF1-REPb and NF1-REPc leading to hemizygosity for a total of 9 protein-coding genes (Fig. 1) (Bengesser et al. 2010; Pasmant et al. 2010; Zickler et al. 2012).

In contrast to type-1, 2 and 3 *NF1* deletions, atypical *NF1* deletions do not have recurrent breakpoints and are heterogeneous in terms of their size and the number of genes located within the deleted region (reviewed by Kehrer-Sawatzki et al. submitted for publication). Approximately 8–10% of all *NF1* microdeletions are considered to be atypical (Pasmant et al. 2010; Messiaen et al. 2011). They may occur as germline deletions but can also be of postzygotic origin and hence may be associated with somatic mosaicism with normal cells (Taylor Tovares et al. 2013). It has been estimated that 59% of atypical *NF1* deletions are of postzygotic origin and thus represent mosaic deletions (Vogt et al. 2014). Atypical *NF1* deletions are caused by a multitude of mutational mechanisms including aberrant DNA double strand break repair, replication-associated errors and retrotransposon-mediated mechanisms (Vogt et al. 2014 and references therein).

Patients with *NF1* microdeletions often exhibit more severe clinical manifestations of NF1 than patients with intragenic pathogenic *NF1* variants and their clinical phenotype has been mainly investigated in patients with type-1 *NF1* deletions (Pasmant et al. 2010; Mautner et al. 2010; Pacot et al. 2021; reviewed by Kehrer-Sawatzki et al. 2017). The lifetime risk of a malignant peripheral nerve sheath tumour (MPNST) in patients with type-1 *NF1* deletions is in the range of 16–26% (De Raedt et al. 2003; Mautner et al. 2010) which is higher than the estimated lifetime risk for an MPNST in all NF1 patients which is 8–15.8% (Evans et al. 2002, 2012; Uusitalo et al. 2016). Further, MPNSTs may occur significantly earlier in patients with *NF1* microdeletions as compared with NF1 patients with intragenic pathogenic variants (De Raedt et al. 2003). Higher numbers of subcutaneous and plexiform neurofibromas and higher growth rates of these tumours have been observed in patients with *NF1* microdeletions as compared to patients with intragenic pathogenic *NF1* variants (Well et al. 2021). In addition, many patients with *NF1* microdeletions exhibit features which are not usually observed in patients with pathogenic variants within the *NF1* gene including facial dysmorphic features, overgrowth, severe global developmental delay and intellectual disability (reviewed by Kehrer-Sawatzki et al. 2017, 2020; Ottenhoff et al. 2020).

It has been postulated that some of the genes co-deleted with *NF1* exert an influence on the clinical manifestation of the disease in patients with *NF1* microdeletions. However, to derive accurate and reliable genotype/phenotype correlations, a precise classification of the types of *NF1* microdeletion is very important since the different types of deletion differ in terms of their extent and hence the number of genes encompassed by the deletion.

In the past, multiplex ligation-dependent probe amplification (MLPA) has been routinely used to identify and characterize *NF1* microdeletions (Wimmer et al. 2006; De Luca et al. 2007). However, there are limitations with regard to the accurate classification of *NF1* microdeletions when MLPA is employed as the only method of analysis. In this review, we outline the utility as well as the limitations of MLPA in relation to the classification of *NF1* microdeletions and discuss alternative methods that may be employed to classify these gross deletions. This is important in the context of establishing genotype/phenotype correlations in patients with *NF1* microdeletions since the different types of *NF1* deletion are associated with the loss of a variable number of genes.

## Classification of *NF1* microdeletions by MLPA

The SALSA^®^ MLPA^®^ Probemix P122-D2 NF1-area (MRC-Holland) includes 35 MLPA-probes. Ten of these are reference probes that detect autosomal regions not located on chromosome 17. Additionally, the probemix contains 23 MLPA-probes that are designed to detect regions located within the chromosomal region 17q11.2 (Table 1). Of the 23 MLPA-probes, 14 map to the type-1 *NF1* microdeletion region (Fig. 1). The probemix also contains two MLPA-probes that map to the short arm of chromosome 17 (17p11.2). A dosage quotient between 0.40 and 0.65 for any given probe is considered to be indicative of a hemizygous deletion. The SALSA^®^ MLPA^®^ Probemix P122-D2 NF1-area is the only commercially available test to identify *NF1* microdeletions by MLPA and it is widely used for clinical diagnostic purposes.

**Table 1:** MLPA results typical for type-1 and type-3 *NF1* microdeletions

Gene (exon)	SALSA MLPA probe designation	Probe position on chromosome 17 (hg19)	MLPA results typical for *NF1* deletion of	
			Type-1	Type-3
*ASPA* (exon 5)^a^	01325-L07456	3397672–3397695	not del	not del
*PMP22* (exon 3)^a^	01463-L00928	15162480–15162457	not del	not del
*TRAF4* (exon 2)	09176-L19109	27074291–27074314	not del	not del
*TRAF4* (exon 4)	08620-L08632	27075052–27075075	not del	not del
*BLMH* (exon 9)	09627-L09912	28599612–28599635	not del	not del
*CPD* (exon 11)	09628-L21977	28770910–28770933	not del	not del
*CPD* (exon 12)	09629-L09914	28789420–28789443	not del	not del
***SUZ12p* (intron 1)**	11798-L12590	29058391–29058414	del	not del
***SUZ12p* (intron 4)**	11801-L12592	29085145–29085168	del	not del
***CRLF3* (exon 3)**	03780-L03289	29124380–29124403	del	not del
***ATAD5* (exon 2)**	03781-L03290	29162044–29162067	del	not del
***ADAP2* (exon)**	03782-L03291	29253873–29253896	del	not del
***RNF135* (exon 2)**	03783-L03292	29311688–29311711	del	not del
***NF1* (exon 1)**	02491-L01922	29421598–29421621	del	del
***NF1* (exon 17)**	02507-L01938	29552202–29552225	del	del
***NF1* (exon 30)**	02512-L01943	29576023–29576046	del	del
***NF1* (exon 48)**	02525-L01956	29676152–29676175	del	del
***NF1* (exon 57)**	05220-L03309	29687576–29687599	del	del
***UTP6* (exon 14)**	03785-L03294	30202348–30202371	del	del
***SUZ12* (exon 10)**	03786-L03295	30315410–30315433	del	del
***LRRC37B* (exon 1)**	03787-L03296	30348569–30348592	del	del
*ZNF207* (exon 9)	09637-L09949	30693753–30693776	not del	not del
*PSMD11* (exon 2)	09632-L09917	30773979–30774002	not del	not del
*MYO1D* (exon 7)	09631-L09916	31094710–31094733	not del	not del
*MYO1D* (exon 2)	09630-L09915	31107652–31107675	not del	not del

The SALSA^®^ MLPA^®^ Probemix P122-D2 NF1-area (MRC-Holland) includes 25 MLPA-probes located on chromosome 17. The sequences corresponding to 14 of these probes are located within the type-1 *NF1* microdeletion interval and are indicated in bold type. If the region covered by these probes is deleted (del), then the deletion is highly likely to represent a type-1 *NF1* deletion harbouring breakpoints within NF1-REPa and NF1-REPc

*not del* not deleted; *del* deleted

^a^These two probes map to 17p11.2. The other probes indicated map to 17q11.2

In the following, both the utility and the accuracy of the MLPA^®^ Probemix P122-D2 NF1-area to classify *NF1* deletions are outlined for each type of deletion.

### Type-1 *NF1* microdeletions

If the target sequences of the 14 MLPA probes shown in red in Fig. 1 are present in only one copy in a patient’s DNA, the *NF1* deletion is considered to be of type-1. Typical MLPA results indicative of a type-1 *NF1* deletion are presented in Table 1. Type-1 *NF1* deletions encompass the region targeted by probe MLPA-probe *LRRC37B* which maps to exon 1 of the *LRRC37B* gene located within NF1-REPc. The genomic region targeted by both MLPA-probes for the *SUZ12P* pseudogene is present in only a single copy in the case of a type-1 *NF1* deletion (Table 1). The MLPA results given in Table 1 are typical for type-1 *NF1* deletions and not for any other type of *NF1* microdeletion. There are, however, considerable distances (269 kb and 345 kb) between the MLPA-probes immediately flanking the type-1 deletion breakpoint regions located within NF1-REPa and NF1-REPc (Fig. 1). In view of the large distances between these MLPA-probes, it may be argued that MLPA is not on its own precise enough to unambiguously classify type-1 *NF1* deletions on the grounds that the deletion breakpoints may not be located within NF1-REPa and NF1-REPc as is characteristic for type-1 deletions. To analyse this in greater detail, Summerer et al. (2018) investigated 236 unselected *NF1* microdeletions which were initially identified by MLPA and presumed to be of type-1 since they encompassed the region targeted by MLPA-probe *LRRC37B* and both MLPA-probes for the *SUZ12P* pseudogene, as indicated in Table 1. Summerer et al. (2018) performed custom-designed array CGH (Agilent SurePrint G3 human CGH microarray) to improve the breakpoint prediction of type-1 *NF1* deletions as well as CytoScan ™ HD array analysis (Affymetrix). The authors precisely identified the deletion breakpoint regions by sequencing breakpoint-spanning PCR products thereby determining the breakpoints at the highest possible resolution. Of the 236 deletions investigated, 234 (99.2%) were classified as bona fide type-1 *NF1* deletions. All 234 deletions had breakpoints located within NF1-REPa and NF1-REPc and were mediated by NAHR, the main mechanism underlying type-1 *NF1* deletions. Only in two of the 236 deletions could the breakpoints not be identified by breakpoint-spanning PCR. Nevertheless, the results of the microarray analysis indicated that the breakpoints of these two deletions were also located within NF1-REPa and NF1-REPc (Summerer et al. 2018). Hence, their analysis of the 236 deletions indicated that *NF1* deletions demarcated by MLPA (as indicated in Table 1) are highly likely to be of type-1. MLPA therefore represents a very efficient method with which to identify type-1 *NF1* deletions. Microarray analysis applied in addition to MLPA could be used to confirm that the deletion breakpoints are indeed located with NF1-REPa and NF-REPc, as is characteristic of type-1 *NF1* deletions. This may be relevant in patients who present with an unusual clinical phenotype which differs from that seen in the majority of patients with type-1 *NF1* deletions.

If patients with type-1 *NF1* deletions present with additional clinical symptoms not frequently encountered in patients with *NF1* microdeletions, the possibility should be considered that additional pathogenic variants might be present in unlinked genes, as recently reported by Santorro et al. (2021). These authors reported a male patient with a type-1 *NF1* deletion and clinical features of the *NF1* microdeletion syndrome that were complicated by cleft palate and other dysmorphic features, hypoplasia of corpus callosum, and partial bicoronal craniosynostosis caused by a novel 2 bp deletion in exon 2 of the Meis homeobox 2 gene (*MEIS2*) inherited from his mildly affected father.

It is important to emphasize that, even using microarray analysis, the breakpoints within NF1-REPa and NF1-REPb cannot be unambiguously assigned owing to the highly repetitive nature of the sequences within the NF1-REPs. Sequences with high homology to the NF1-REPs are present in multiple copies on chromosome 17 (Giannuzzi et al. 2013). Owing to the repetitivity of these sequences, the breakpoints cannot be precisely detected by microarray analysis with standard software tools used to analyse germline chromosomal aberrations (Summerer et al. 2018). The breakpoints of type-1 deletions can only be narrowed down by means of breakpoint-spanning PCRs and sequence analysis of the respective PCR products (Hillmer et al. 2017; Summerer et al. 2018). Of the 236 type-1 *NF1* deletions analysed by Summerer et al. (2018), 179 (75.8%) harboured breakpoints within the NAHR hotspot PRS2 which spans 4.8 kb. By contrast, 39 (16.5%) type-1 deletions had breakpoints within PRS1 encompassing 5.2 kb. Most of the remaining 18 deletions exhibited breakpoints that were located between PRS1 and PRS2 within a genomic region of 14 kb exhibiting high sequence similarity between NF1-REPa and NF1-REPc. In total, 13 (5.5%) of the 236 type-1 deletions analysed had breakpoints within this 14 kb region (Summerer et al. 2018).

### Type-3 *NF1* deletions

Only 8 type-3 *NF1* deletions have been identified so far by means of accurate breakpoint analysis (Bengesser et al. 2010; Pasmant et al. 2010; Zickler et al. 2012). All of them exhibited the same MLPA results as indicated in Table 1. By means of deletion breakpoint-spanning PCR and sequence analysis of the PCR-products, the breakpoints of these 8 type-3 *NF1* deletions were identified within homologous regions between NF1-REPb and NF1-REPc indicative of NAHR as the underlying mechanism. It follows that type-3 *NF1* deletions ascertained using MLPA, as indicated in Table 1, are likely to be *bona fide*. However, owing to the large distances between the MLPA-probes flanking the breakpoint regions, additional tools, such as breakpoint-spanning PCRs or microarray analysis, are required in order to finally confirm the presence of a *bona fide* type-3 deletion.

Zhang et al. (2015) identified a large *NF1* deletion in patient NF073 with the same MLPA-probe pattern as that observed for the 8 type-3 *NF1* deletions mentioned above. The deletion of patient NF073 was classified as atypical by Zhang et al. (2015). However, since MLPA was the only analytical method performed, an unambiguous distinction between type-3 and atypical deletion could not be made. To distinguish between both types of *NF1* deletion would be important in terms of assessing the number of genes encompassed by the deletion, the likelihood of somatic mosaicism with normal cells, as well as the mutational mechanism underlying the corresponding deletion.

### Type-2 *NF1* deletions

Type-2 deletions cannot be classified with any degree of accuracy by employing MLPA as the single method of analysis. This conclusion may be drawn from the findings of Vogt et al. (2012, 2014) who analysed type-2 as well as atypical *NF1* deletions and compared the corresponding MLPA results. In all, Vogt et al. analysed 40 type-2 *NF1* deletions with breakpoints located within *SUZ12* and *SUZ12P* as confirmed by breakpoint-spanning PCRs. A breakpoint localization within *SUZ12* and *SUZ12P* is characteristic of type-2 deletions. The 40 type-2 deletions analysed by Vogt et al. (2012) exhibited breakpoints within sequences homologous between *SUZ12* and *SUZ12P,* which is indicative of NAHR being the major causative mechanism underlying these deletions (Vogt et al. 2012). Two different MLPA results were obtained for these 40 type-2 *NF1* deletions (Table 2). Nine of the 40 deletions encompassed the region corresponding to MLPA-probe *SUZ12P* intron 4. By contrast, 31 of the 40 type-2 *NF1* deletions did not include the region corresponding to this MLPA-probe (Table 2). None of the 40 type-2 deletions analysed by Vogt et al. (2012) encompassed the region targeted by MLPA-probe *SUZ12* exon 10, which is not located within the region of sequence homology between *SUZ12* and *SUZ12P*.

**Table 2:** MLPA results observed in 40 type-2 *NF1* deletions and 9 atypical *NF1* deletions (group #2A deletions)

Gene (exon)	SALSA MLPA probe designation	Probe position on chromosome 17 (hg19)	Type-2 deletions		Patient ID of the 9 atypical group #2A *NF1* deletions								
			(*N = 9*)^a^	(*N* = 31)^a^	D1008345^a,b^	2535^b^	R84329^a,b^	R48018^a,b^	Ak-47055^b^	R97108^a^	R49005^a^	#4^c^	556^d^
*ASPA* (exon 5)	01325-L07456	3397672–3397695	not del	not del	not del	not del	not del	not del	not del	not del	not del	not del	not del
*PMP22* (exon 3)	01463-L00928	15162480–15162457	not del	not del	not del	not del	not del	not del	not del	not del	not del	not del	not del
*TRAF4* (exon 2)	09176-L19109	27074291–27074314	not del	not del	not del	not del	not del	not del	not del	not del	not del	not del	not del
*TRAF4* (exon 4)	08620-L08632	27075052–27075075	not del	not del	not del	not del	not del	not del	not del	not del	not del	not del	not del
*BLMH* (exon 9)	09,627-L09912	28,599,612–28,599,635	not del	not del	not del	not del	not del	not del	not del	not del	not del	not del	not del
*CPD* (exon 11)	09628-L21977	28770910–28770933	not del	not del	not del	not del	not del	not del	not del	not del	not del	not del	not del
*CPD* (exon 12)	09629-L09914	28789420–28789443	not del	not del	not del	not del	not del	not del	not del	not del	not del	not del	not del
*SUZ12P* (intron 1)	11798-L12590	29058391–29058414	not del	not del	not del	not del	not del	not del	not del	not del	not del	not del	not del
*SUZ12P* (intron 4)	11801-L12592	29085145–29085168	del	not del	not del	not del	del	del	del	not del	del	not del	not del
*CRLF3* (exon 3)	03780-L03289	29124380–29124403	del	del	del	del	del	del	del	del	del	del	del
*ATAD5* (exon 2)	03781-L03290	29162044–29162067	del	del	del	del	del	del	del	del	del	del	del
*ADAP2* (exon)	03,782-L03291	29253873–29253896	del	del	del	del	del	del	del	del	del	del	del
*RNF135* (exon 2)	03783-L03292	29311688–29311711	del	del	del	del	del	del	del	del	del	del	del
*NF1* (exon 1)	02491-L01922	29421598–29421621	del	del	del	del	del	del	del	del	del	del	del
*NF1* (exon 17)	02507-L01938	29552202–29552225	del	del	del	del	del	del	del	del	del	del	del
*NF1* (exon 30)	02512-L01943	29576023–29576046	del	del	del	del	del	del	del	del	del	del	del
*NF1* (exon 48)	02525-L01956	29,676,152–29,676,175	del	del	del	del	del	del	del	del	del	del	del
*NF1* (exon 57)	05220-L03309	29687576–29687599	del	del	del	del	del	del	del	del	del	del	del
*UTP6* (exon 14)	03785-L03294	30202348–30202371	del	del	del	del	del	del	del	del	del	del	del
*SUZ12* (exon 10)	03786-L03295	30315410–30,315,433	not del	not del	not del	not del	not del	not del	not del	not del	not del	not del	not del
*LRRC37B* (exon 1)	03787-L03296	30348569–30348592	not del	not del	not del	not del	not del	not del	not del	not del	not del	not del	not del
*ZNF207* (exon 9)	09637-L09949	30693753–30693776	not del	not del	not del	not del	not del	not del	not del	not del	not del	not del	not del
*PSMD11* (exon 2)	09632-L09917	30773979–30774002	not del	not del	not del	not del	not del	not del	not del	not del	not del	not del	not del
*MYO1D* (exon 7)	09631-L09916	31094710–31094733	not del	not del	not del	not del	not del	not del	not del	not del	not del	not del	not del
*MYO1D* (exon 2)	09630-L09915	31107652–31107675	not del	not del	not del	not del	not del	not del	not del	not del	not del	not del	not del

Importantly, all deletions of type-2 as well as the 9 atypical *NF1* deletions of group #2A do not include the region covered by the *SUZ12* MLPA-probe of the SALSA MLPA-probemix P122, version D2. From the MLPA results, type-2 deletions cannot be distinguished from atypical *NF1* deletions of group #2A

*not del* not deleted; *del* deleted

^a^The 40 type-2 *NF1* deletions and five atypical *NF1* deletions of the patients indicated were analysed by Vogt et al. (2012)

^b^The five atypical deletions were analysed by Vogt et al. (2014)

^c^The deletion of patient #4 was analysed by Parisien-La Salle et al. (2019)

^d^The deletion of patient 556 was characterized by Büki et al. (2021)

However, the MLPA results observed in the 40 type-2 *NF1* deletions analysed by Vogt et al. (2012) are by no means exclusive to type-2 *NF1* deletions. Vogt et al. (2012, 2014) identified 9 of 19 atypical *NF1* deletions exhibiting the same MLPA results as those observed for type-2 *NF1* deletions. As determined by breakpoint-spanning PCR or custom-designed MLPA, the breakpoints of these 9 atypical *NF1* deletions were not located within homologous regions between *SUZ12* and *SUZ12P* as is characteristic for type-2 deletions. Instead, the proximal breakpoints of these 9 deletions were located either within *SUZ12P, CRLF3* or between *SUZ12P* and *CRLF3*. The telomeric deletion breakpoints were located either within the *UPT6* gene or the genomic regions between *UTP6* and *SUZ12* (Table 3). The molecular mechanism responsible for these atypical *NF1* deletions was not NAHR but instead non-homologous end joining (NHEJ) or a replication-based mechanism (Vogt et al. 2014). In the following, these atypical *NF1* deletions are termed group #2A deletions.

**Table 3:** Breakpoint locations of the 9 atypical *NF1* deletions (group #2A deletions) which cannot be distinguished from type-2 *NF1* deletions by means of MLPA

Patient	Breakpoint locations	Deletion size	Centromeric breakpoint location	Telomeric breakpoint location
D1008345	29,094,424 (30,218,204)^a^	1,123,781 bp	*SUZ12P*	*UTP6*
2535	29,101,686 (30,250,762)^a^	1,149,077 bp	*SUZ12P*	Between *UTP6* and *SUZ12*
R84329	29,074,557 (30,223,384)^a^	1,148,828 bp	*SUZ12P*	*UTP6*
R48018	29,084,006 (30,241,383)^a^	1,157,378 bp	*SUZ12P*	Between *UTP6* and *SUZ12*
Ak-47055	29,082,023 (30,243,011)^a^	1,160,989 bp	*SUZ12P*	Between *UTP6* and *SUZ12*
R97108	29,098,365–29,107,598^b^ (30,202,371–30,250,614)	1.1–1.2 Mb	*SUZ12P*	Between *UTP6* and *SUZ12*
R49005	29,058,862–29,068,410^b^ (30,202,371–30,250,614)	1.1–1.2 Mb	*SUZ12P*	Between *UTP6* and *SUZ12*
#4	29,116,494 (30,260,501)^c^	1,144,007 bp	*CRLF3*	Between *UTP6* and *SUZ12*
556	29,100,044–29,104,296^d^ (30,226,743–30,227,597)	1,122,447 bp	*Between SUZ12P and CRLF3*	*UTP6*

Indicated are the genomic positions of the centromeric breakpoints and, in parentheses, the positions of the telomeric breakpoints according to the human genome version GRCh/hg19

^a^Genomic positions correspond to the nucleotides immediately before and immediately after the deleted DNA sequence. The deletion breakpoints were identified by breakpoint-spanning PCRs and sequence analysis of these PCR products (Vogt et al. 2014)

^b^The breakpoint regions as determined by custom-designed MLPA (Vogt et al. 2012)

^c^The breakpoints as determined by microarray analysis (Parisien-La Salle et al. 2019)

^d^The breakpoint regions as determined by microarray analysis (Büki et al. 2021)

According to the findings of Vogt et al. (2012, 2014), type-2 *NF1* deletions and atypical group #2A deletions cannot be distinguished from one another by MLPA. Other techniques such as microarray analysis, specifically tailored to detect chromosomal aberrations at high resolution [as performed by Pasmant et al. (2009), Vogt et al. (2014) and Summerer et al. (2019)] or sequence analysis of breakpoint-spanning PCRs, have to be employed to distinguish between these types of *NF1* deletion. This may well be of clinical significance since *SUZ12* is functionally inactivated by the breakpoints of type-2 deletions. *SUZ12* inactivation by intragenic pathogenic variants has been shown to cause overgrowth, dysmorphic features, musculoskeletal abnormalities and developmental delay/intellectual disability (Imagawa et al. 2018; Cyrus et al. 2019a, b). By contrast, *SUZ12* is present in two copies and is not affected by the breakpoints of atypical *NF1* deletions of group #2A.

NAHR between *SUZ12* and *SUZ12P* is the major mechanism underlying type-2 *NF1* deletions. However, rare cases of type-2 deletions have been reported that exhibit breakpoints at non-homologous sites within *SUZ12* and *SUZ12P* and hence are not mediated by NAHR (Vogt et al. 2012). These deletions could not be distinguished from type-2 deletions mediated by NAHR if MLPA using the SALSA® MLPA® Probemix P122-D2 NF1-area were the only analytical method employed to characterize these deletions. Microarray-analysis using targeted arrays, custom-designed MLPA analysis and breakpoint-spanning PCRs would be necessary to narrow down the breakpoints of type-2 deletions and distinguish between those mediated by NAHR and those caused by other mutational mechanisms (Vogt et al. 2012).

### Atypical *NF1* deletions

As yet, a total of 61 atypical *NF1* deletions have been reported in the literature (reviewed by Kehrer-Sawatzki et al. submitted for publication). In contrast to the *NF1* deletions mediated by NAHR, atypical *NF1* deletions do not exhibit recurrent breakpoints and are quite heterogeneous in terms of their size and the number of genes located within the deletion regions. Of the 61 known atypical *NF1* deletions reported so far, 31 exhibit breakpoints which are located beyond one or both boundaries of the type-1 *NF1* deletions. Of these 31 deletions, 27 are larger than 1.4-Mb and encompass more than the 14 protein-coding genes located within the type-1 *NF1* deletion region (reviewed by Kehrer-Sawatzki et al. submitted for publication). Hence, these large deletions are likely to be of lesser importance in terms of genotype/phenotype correlations since the loss of additional genes located beyond the boundaries of the type-1 *NF1* microdeletion region probably gives rise to an even more complex clinical phenotype and may be associated with additional clinical features not frequently observed in patients with type-1 *NF1* deletions. In any case, these large atypical *NF1* deletions can be detected by MLPA but neither the precise breakpoints nor the exact number of genes located within the deletion region can be ascertained using MLPA as the sole method of analysis. Owing to the limited number of MLPA-probes included in the SALSA® MLPA® Probemix P122-D2 NF1-area, the accurate characterization of these deletions in terms of their extent is not possible by MLPA, and only feasible by a method such as microarray analysis that is well suited to detect chromosomal aberrations at high resolution (Table 4).

**Table 4:** Types of *NF1* microdeletion and the methods required to classify them to be able to perform genotype/phenotype correlations

*NF1* microdeletion type (frequency)	Methods needed to classify the deletion unambiguously and to determine the number of genes deleted	Frequency of somatic mosaicism
Type-1 (70–80%)	MLPA® Probemix P122-D2 NF1-area or microarray analysis^**a**^	Very rare
Type-2 (~ 10%)	Microarray analysis^a^	At least 70%^b^
Type-3 (1–4%)	Microarray analysis^**a**^	Unknown
Atypical (10–20%)	Microarray analysis^a^	At least 59%^c^

The estimated frequency of somatic mosaicism with normal cells associated with each deletion type is indicated

^a^High-resolution microarray analysis is recommended by means of e.g. the Cytoscan high-density (HD) SNP-array (Affymetrix) or custom-designed targeted arrays (Agilent Technologies) for the high resolution of individual breakpoints [as performed by Pasmant et al. (2009), Vogt et al. (2014) and Summerer et al. (2018)]

^b^According to Steinmann et al. (2007) and Messiaen et al. (2011)

^c^According to Vogt et al. (2014)

**Fig. 2:**
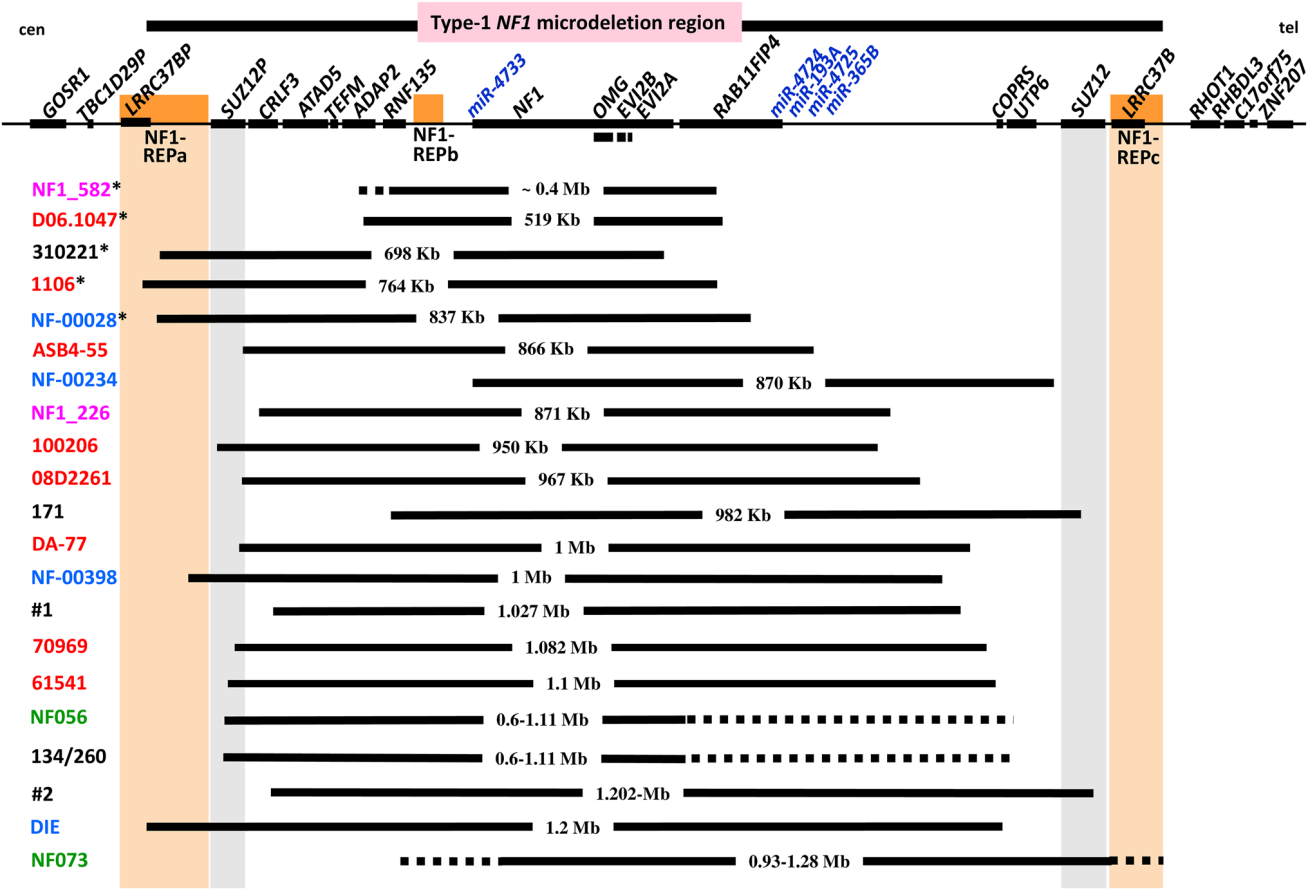
Schema of the type-1 *NF1* microdeletion region, which includes 14 protein-coding genes as well as the *SUZ12P* pseudogene and 5 microRNA genes. The relative locations of these genes are indicated by black rectangles. Indicated below is the extent of the 21 known atypical group #2 *NF1* deletions represented by vertical black bars. The patient IDs are indicated on the left. The low-copy repeats, NF1-REPa and NF1-REPc, are located at the boundaries of the type-1 *NF1* microdeletion region. The atypical *NF1* deletions of group #2, which exhibit breakpoints located within the boundaries of the type-1 *NF1* microdeletion region, are smaller than type-1 *NF1* deletions and do not encompass all of the genes located within the type-1 *NF1* microdeletion region. As yet, 30 atypical group #2 *NF1* deletions have been reported; indicated are 21 of these atypical group #2 deletions which can be distinguished from type-1 *NF1* deletions by MLPA. Patients with IDs indicated in red were analysed by Vogt et al. (2012, 2014), those indicated in blue were analysed by Pasmant et al. (2010), in pink by Bianchessi et al. (2015) and in green by Zhang et al. (2015). Patient 171 was analysed by Ferrari et al. (2017), patients #1 and #2 by Serra et al. (2019), patients 134/260 by Büki et al. (2021) and patient 310221 by Kehrer-Sawatzki et al. submitted for publication). The deletions of the 5 patients whose IDs are marked by an asterisk do not encompass 4 of the 5 microRNA genes located within the type-1 *NF1* microdeletion region. *cen* centromeric; *tel* telomeric

**Fig. 3:**
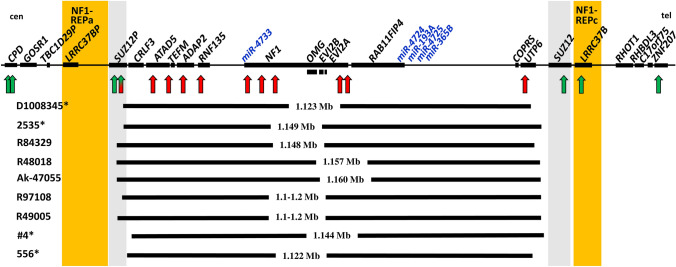
Schema of the type-1 *NF1* microdeletion region, which includes 14 protein-coding genes as well as the *SUZ12P* pseudogene and 5 microRNA genes. The relative locations of these genes are indicated by black rectangles. Indicated below is the extent of the 9 known atypical group #2A *NF1* deletions which are represented by vertical black bars. Patient IDs are indicated on the left. The low-copy repeats, NF1-REPa and NF1-REPc, are located at the boundaries of the type-1 *NF1* microdeletion region. The atypical *NF1* deletions of group #2A, which exhibit breakpoints located within the boundaries of the type-1 *NF1* microdeletion region, cannot be distinguished from type-2 deletions using MLPA. The vertical red and green arrows represent the binding sites of the MLPA-probes included in the SALSA^®^ MLPA^®^ Probemix P122-D2 NF1-area (MRC-Holland). Red arrows represent MLPA-probes targeting genomic regions encompassed by the respective heterozygous deletions whereas green arrows represent MLPA-probes targeted to regions which are not deleted and present in two copies. The MLPA-probe *SUZ12P* intron 4 is shaded in green/red because the region targeted by this probe is deleted only in the patients marked by an asterisk, and not in the other 5 atypical group #2A deletions which are depicted here. cen: centromeric; tel: telomeric

In contrast to the 31 atypical *NF1* deletions with breakpoints located beyond one or both boundaries of the type-1 *NF1* deletions, a second group of atypical *NF1* deletions has been identified which is characterized by breakpoints located within the type-1 *NF1* microdeletion region (Pasmant et al. 2010; Vogt et al. 2012, 2014; Bianchessi et al. 2015; Zhang et al. 2015; Ferrari et al. 2017; Parisien-La Salle et al. 2019; Serra et al. 2019; Büki et al. 2021; Kehrer-Sawatzki et al. submitted for publication). These deletions are shorter than type-1 deletions and encompass only a subset of the 14 protein-coding genes located within the type-1 *NF1* deletion region (Figs. 2 and 3). These deletions have been termed atypical group #2 deletions (Kehrer-Sawatzki et al. submitted for publication). So far, 30 atypical group #2 *NF1* deletions have been identified (Figs. 2 and 3). By means of MLPA, these deletions can be distinguished from type-1, and in most instances also from type-3 deletions. Of these 30 atypical deletions, 21 can be also distinguished from type-2 deletions using MLPA as a single method of analysis. The relative extents of these 21 atypical deletions are indicated in Fig. 2. However, 9 of the 30 deletions cannot be distinguished from type-2 deletions by means of MLPA (Table 2). These 9 deletions represent a subgroup of group #2 deletions and are termed group #2A deletions. Their deletion boundaries are schematically indicated in Fig. 3. Breakpoint analysis revealed that the breakpoints of these 9 deletions are not located within *SUZ12P* and *SUZ12* as would be characteristic of type-2 deletions (Vogt et al. 2012, 2014; Parisien-La Salle et al. 2019; Büki et al. 2021) (Table 3). Thus, atypical group #2A deletions and type-2 *NF1* deletions cannot be distinguished from one another by MLPA. They do, however, differ from one another in terms of the genes they encompass. None of the 9 atypical group #2A deletions includes the *SUZ12* gene. By contrast, the telomeric breakpoints of type-2 deletions are located within *SUZ12* which is thereby functionally inactivated.

Taken together, 9 (30%) of the 30 atypical deletions of group 2 cannot be accurately classified by MLPA. This is particularly relevant in view of the fact that the atypical group #2A *NF1* deletions represent those deletions which are most important in the context of genotype/phenotype correlations since they may encompass only a subset of the 14 protein-coding and 5 microRNA genes located within the type-1 *NF1* deletion interval.

## Discussion

Patients with type-1 *NF1* deletions often exhibit a severe clinical phenotype characterized by features that are not frequently seen in patients with intragenic pathogenic *NF1* variants such as dysmorphic facial features, severe global developmental delay, cognitive disability, increased MPNST risk and a high number (as well as an accelerated growth rate) of neurofibromas (reviewed by Kehrer-Sawatzki et al. 2017, 2020; Ottenhoff et al. 2020; Büki et al. 2021; Pasmant et al. 2021; Pacot et al. 2021; Well et al. 2021). Genes located within the type-1 *NF1* deletion interval and co-deleted with *NF1* are likely to be responsible for the severe *NF1* microdeletion-associated phenotype giving rise to the *NF1* microdeletion syndrome. Importantly, the vast majority of *NF1* microdeletion patients clinically characterized to date had a type-1 *NF1* deletion, which is the most common type of *NF1* deletion, observed in 70–80% of *NF1* deletion patients.

Considerably less is known about the clinical phenotype in patients with other types of *NF1* deletion. This may be due to the fact that these deletions occur much less frequently than type-1 *NF1* deletions. Furthermore, type-2 *NF1* deletions as well as atypical *NF1* deletions are more frequently associated with somatic mosaicism with normal cells lacking the deletion; this may lead to a milder form of the disease. Somatic mosaicism is likely to be associated with highly variable clinical consequences depending not only on the *NF1* microdeletion type, but also on the developmental stage when it has arisen, the cell types involved and the proportion of cells affected. This will certainly limit our ability to relate the clinical phenotype to the mutant genotype. To date, only 5 patients with non-mosaic type-2 *NF1* deletions have been clinically characterized (Vogt et al. 2011; Zhang et al. 2015; Büki et al. 2021; Yethindra et al. 2021). From these data, it may be concluded that non-mosaic type-2 *NF1* deletions are associated with a severe clinical phenotype similar to that exhibited by patients with type-1 *NF1* deletions. However, further studies involving additional patients with non-mosaic type-2 deletions, accurately classified by methods with higher resolution than MLPA, would be necessary to confirm this.

By the same token, only a few patients with atypical *NF1* deletions shorter than 1.4-Mb have been clinically characterized in any detail). The paucity of such patients has hampered the establishment of genotype/phenotype correlations. Patients with atypical *NF1* deletions that do not encompass all of the genes located within the type-1 *NF1* microdeletion interval may be very informative in terms of identifying potential modifier genes that could contribute to the severe phenotype observed in type-1 *NF1* deletions. The clinical data available for 6 patients with such atypical group #2 deletions may provide support for this postulate since they manifested a less severe phenotype than that observed in patients with type-1 *NF1* deletions (Kehrer-Sawatzki et al. submitted for publication).

To establish meaningful genotype/phenotype correlations, an accurate classification of *NF1* microdeletions is crucial since the different types of *NF1* deletion are associated with the loss of different numbers of flanking genes. The type-1 *NF1* microdeletion region encompasses 14 protein-coding genes and 5 microRNA genes (miR-4733, miR-4724, miR-193A, miR-4725 and miR-365B). Whilst type-1, 2 and 3 deletions are invariably associated with the loss of all five microRNA genes, some atypical *NF1* deletions do not include all five (Fig. 2). Hemizygosity for some of these microRNA genes may well be of clinical importance. The best characterized of the five microRNA genes within the *NF1* gene region is miR-193A which is known to possess tumour suppressor functions (Jin et al. 2019; Chen et al. 2020; Polini et al. 2020; Wei et al. 2021). Hemizygosity for this microRNA gene may therefore facilitate tumour growth in those patients with *NF1* microdeletions that encompass it. Five of the 30 atypical *NF1* deletions of group #2 analysed to date do not include miR-193A (Fig. 2). However, the patients harbouring these deletions were either very young or clinically not characterized in any detail and hence it would be premature to discuss numbers of neurofibromas and their growth rates.

Five of the 14 protein-coding genes located within the type-1 *NF1* deletion region, *ATAD5, NF1, OMGP, RAB11FIP4* and *SUZ12,* are suspected to be loss-of-function intolerant since they exhibit ‘probability of loss-of-function” (pLI) scores of 0.99 or 1.0 (Supp. Table S1) (Lek et al. 2016). Consequently, hemizygosity for these genes is highly likely to exert a detrimental impact on the clinical phenotype in patients with deletions that encompass these genes. The different types of *NF1* microdeletion are associated with variable copy numbers of these genes (Supp. Table S1). The *SUZ12* gene, with a pLI score of 1.0, is very likely to be an important modifier of the type-1 *NF1* microdeletion-associated phenotype. In patients with *NF1* microdeletions, the loss of *SUZ12* has been shown to increase the risk of MPNSTs (De Raedt et al. 2014; Lee et al. 2014; Zhang et al. 2014). Further, biallelic loss of *SUZ12* is important in MPNST tumorigenesis (De Raedt et al. 2014). Patients with pathogenic variants located within *SUZ12* but without *NF1* microdeletions exhibit overgrowth, facial dysmorphic features, musculoskeletal abnormalities and developmental delay/intellectual disability (Imagawa et al. 2018; Cyrus et al. 2019a, b; Choufani et al. 2020). Thus, the loss of *SUZ12* is likely to contribute significantly to the facial dysmorphic features, overgrowth, severe global developmental delay and the cognitive disabilities which are frequently noted in patients with type-1 *NF1* deletions or other types of *NF1* microdeletion that encompass the suppressor of zeste (*SUZ12*) gene. These clinical features are not observed in patients with intragenic pathogenic *NF1* variants who possess two functional copies of the *SUZ12* gene. Hence, the loss of *SUZ12* in patients with *NF1* microdeletions is most likely causally associated with these clinical features. This conclusion is supported by the clinical phenotype of patients with atypical group #2 deletions who are not hemizygous for a *SUZ12* deletion (Kehrer-Sawatzki et al. submitted for publication). However, only 6 of these patients have been clinically characterized to date and further studies are necessary to corroborate this putative genotype/phenotype correlation.

A specific role in the development of the *NF1* microdeletion-associated phenotype has recently been demonstrated for the cytokine receptor-like factor 3 (*CRLF3*) gene located in the centromeric part of the *NF1* microdeletion region (Fig. 1). Induced pluripotent stem cell-forebrain cerebral organoids (hCOs), isolated from patients with type-1 *NF1* microdeletions, display both neural stem cell proliferation and elevated neuronal abnormalities such as dendritic maturation deficits. Whilst increased neuronal stem cell proliferation has been shown to result from decreased NF1/RAS regulation, the neuronal differentiation, survival and maturation defects of these hCOs are caused by reduced *CRLF3* expression and impaired RhoA signalling (Wegscheid et al. 2021). This role of *CRLF3* has been corroborated by the observation that hCOs, isolated from a patient with an atypical *NF1* deletion not encompassing the *CRLF3* gene, did not exhibit abnormalities of neuronal survival, differentiation and maturation (Wegscheid et al. 2021). Further, these authors identified 7 of 17 NF1 patients with an increased autistic trait burden who harboured a germline missense putatively pathogenic variant within the *CRLF3* gene (c.1166T>C, p.Leu389Pro) present in addition to pathogenic variants in the *NF1* gene. Taken together, these findings indicate an essential role for *CRLF*3 in both human brain development and autism (Wegscheid et al. 2021). Indeed, a high autistic trait burden has been observed in children with type-1 *NF1* deletions associated with the loss of one *CRLF3* gene copy (Kehrer-Sawatzki et al. 2020). As yet, the clinical phenotype of patients with atypical *NF1* deletions that do not encompass the *CRLF3* gene has not been characterized in any detail with regard to the presence or absence of autistic traits. Further analyses of patients with deletions of this type will be necessary to determine the contribution of the *CRLF3* gene to the *NF1* microdeletion-associated phenotype.

The MLPA® Probemix P122-D2 NF1-area has turned out to be a valuable means to identify *NF1* microdeletions and to characterize them, at least to a certain extent. A classification of *NF1* deletions by MLPA is possible for type-1 deletions as shown by Summerer et al. (2018). According to their analysis, 99% of *NF1* deletions initially identified by MLPA were indeed type-1 *NF1* deletions as determined by sequence analysis of the breakpoints. By contrast, type-2 *NF1* deletions and certain atypical *NF1* deletions, those of group #2A, cannot be distinguished by MLPA; thus, further higher resolution analytical methods such as microarray analysis are required to determine the breakpoints and hence the number of genes included in the corresponding deletion intervals. The precise extent of *NF1* deletions is critically important for establishing genotype/phenotype correlations and identifying potential modifier genes. Guidelines indicating the appropriate methods to use for the accurate classification of the different types of *NF1* microdeletion are presented in Table 4. Correct classification of the deletion type is a prerequisite for being able to predict the potential presence of somatic mosaicism with normal cells. Type-1 *NF1* deletions are only very rarely mosaic and the vast majority are germline deletions (Messiaen et al. 2011; Summerer et al. 2019). This is in accordance with the analysis of the parental origin of these deletions and their underlying mutational mechanism. It has been shown that 71% of type-1 *NF1* microdeletions are caused by interchromosomal unequal crossover between either maternal or paternal chromosomes which confirms that these deletions are predominantly of meiotic origin. In most instances, type-1 *NF1* deletions arise in the maternal germline (Lopez-Correa et al. 2000; Neuhäusler et al. 2018).

By contrast, somatic mosaicism is frequent in patients with type-2 and atypical *NF1* deletions (Steinmann et al. 2007; Messiaen et al. 2011; Roehl et al. 2012; Vogt et al. 2014) (Table 4). Indeed, it has been estimated that at least 70% of all type-2 *NF1* deletions are of postzygotic origin (Steinmann et al. 2007; Messiaen et al. 2011). So far, only 5 patients with non-mosaic type-2 *NF1* deletions have been reported (Vogt et al. 2011; Zhang et al. 2015; Büki et al. 2021; Yethindra et al. 2021). The reason why the paralogous sequences *SUZ12* and *SUZ12P* are more often involved in mitotic NAHR and only rarely in meiotic NAHR giving rise to non-mosaic type-2 *NF1* deletions, are unclear. Genomic regions of increased NAHR activity giving rise to chromosomal aberrations have been reported to experience frequent allelic homologous recombination (AHR) during meiosis (Torres-Juan et al. 2007). Further, both the NAHR hotspots PRS1 and PRS2, located within the NF1-REPs, and those located within the CMT1A-REPs, overlap with pre-existing AHR hotspots (De Raedt et al. 2006; Lindsay et al. 2006). By contrast, the meiotic AHR activity within the region of *SUZ12* and *SUZ12P* is low (Mussotter et al. 2014) which may explain the relatively rare occurrence of meiotic NAHR events causing non-mosaic type-2 *NF1* deletions.

Mosaicism with normal cells has been shown to influence the clinical phenotype in patients with *NF1* microdeletions, often leading to a mild manifestation of the disease (Kehrer-Sawatzki et al. 2012; Taylor Tovares et al. 2013). Hence the accurate classification of the type of *NF1* microdeletion by methods such as microarray analysis may also help to identify patients with a high probability of being mosaic which might then require analysis of tissues other than blood in order to confirm or exclude mosaicism (Table 4). The assessment of mosaicism with normal cells without the deletion especially in founder patients carrying an *NF1* microdeletion other than type 1 is very important with respect to deriving genotype/phenotype correlations. This assessment requires methodologies other than MLPA or microarray-analysis that are able to determine cells with and without the deletion at high resolution. Low-grade mosaicism with normal cells may be overlooked in patients with *NF1* deletions investigated by MLPA as the single method of analysis since the intrinsic detection limit of mosaicism is in the range of 10–20% (reviewed by Summerer et al. 2019). If patients have high proportions of cells with the *NF1* microdeletion in their blood, normal cells present at proportions lower than 10–20% are not going to be detectable by MLPA. Similar detection limits are associated with other methods such as microarray analysis and Sanger sequencing (reviewed by Summerer et al. 2019). Therefore, quantitative methods are necessary to detect or exclude low-grade mosaicism in patients with *NF1* microdeletions, e.g. quantitative PCR (qPCR), droplet digital PCR (ddPCR), deep next-generation sequencing and fluorescence in situ hybridization (FISH) on a large number of cells. These methods, performed using different cell types such as blood lymphocytes and skin fibroblasts, may reliably establish whether the *NF1* microdeletion is mosaic or not, which is important not only in relation to the observed phenotype in the patient, but also for the anticipated severity of the disease in the next generation, and the recurrence risk for siblings.

## Conclusion

MLPA is a valuable method for the identification of large *NF1* deletions but it has its limitations with regard to the accurate classification of the different *NF1* microdeletion types. Whereas type-1 *NF1* deletions appear to be classified relatively precisely by MLPA, this method fails to distinguish between type-2 and atypical group #2A *NF1* deletions which represent 30% of all atypical *NF1* deletions with breakpoints located within the type-1 *NF1* microdeletion region (atypical *NF1* deletions of group #2). Patients with atypical group #2 *NF1* deletions may facilitate the establishment of genotype/phenotype correlations if they are associated with hemizygosity for only a subgroup of the genes located within the type-1 *NF1* microdeletion interval. The precise characterization of these deletions in terms of their extent is a prerequisite for identifying such correlations.

## Supplementary Information

Below is the link to the electronic supplementary material.

Supplementary file1 (DOCX 16 KB)

## Data Availability

The manuscript does not have associated data.

## Code availability

The manuscript does not include software application or custom codes.
