# Supplementary material for: Classification of NF1 microdeletions and its importance for establishing genotype/phenotype correlations in patients with NF1 microdeletions
Source: Hum Genet. 2021 Sep 18;140(12):1635–49. doi: 10.1007/s00439-021-02363-3 (PMC8553723; doi:10.1007/s00439-021-02363-3)
Supplement: Supplementary file 1 — Supplementary file1 (DOCX 16 KB) [file 439_2021_2363_MOESM1_ESM.docx]

**Supplementary Table S1:** Protein-coding genes located within the 1.4-Mb spanning type-1 *NF1* microdeletion region and their ‘probability of loss-of-function’ (pLI) score.

| Gene | MIM# | pLI-score | Copy number of the gene in deletions of | | | |
| --- | --- | --- | --- | --- | --- | --- |
|  |  |  | type-1 | type-2 | type-3 | atypical #2A |
| *CRLF3* | 614853 | 0 | 1 | 1 | 2 | 1 |
| *ATAD5* | 609534 | 1.00 | 1 | 1 | 2 | 1 |
| *TEFM* | 616422 | 0.51 | 1 | 1 | 2 | 1 |
| *ADAP2* | 608635 | 0.00 | 1 | 1 | 2 | 1 |
| *RNF135* | 611358 | 0.00 | 1 | 1 | 2 | 1 |
| *NF1* | 162200 | 1.00 | 1 | 1 | 1 | 1 |
| *OMG* | 164345 | 0.97 | 1 | 1 | 1 | 1 |
| *EVI2B* | 158381 | 0.06 | 1 | 1 | 1 | 1 |
| *EVI2A* | 158380 | 0.00 | 1 | 1 | 1 | 1 |
| *RAB11FIP4* | 611999 | 0.99 | 1 | 1 | 1 | 1 |
| *COPRS* | 616477 | 0.25 | 1 | 1 | 1 | 1 |
| *UTP6* | ̶ | 0 | 1 | 1 | 1 | 1 or 2 |
| *SUZ12* | 613675 | 1.00 | 1 | 2 | 1 | 2 |
| *LRRC37B* | 616558 | 0.01 | 1 | 2 | 1 | 2 |

The gnomAD browser (<https://gnomad.broadinstitute.org/>) indicates the constraint metric “probability of loss-of-function” (pLI) which considers the observed and expected variant counts for a gene. A pLI score ≥ 0.9 is indicative of genes that are predicted to be intolerant of loss-of-function variants (Lek et al. 2016). The pLI-score indicated is according to gnomAD v2.1.1/ gnomAD SVs v2.1.
